# Adaptive Family Responses to Dementia Care: A Four-Year Longitudinal Study in Hong Kong

**DOI:** 10.1093/geroni/igaf122.4059

**Published:** 2025-12-31

**Authors:** Jacky C P Choy, Gloria Hoi Yan Wong, Cheng Shi

**Affiliations:** The University of Hong Kong, Hong Kong, Hong Kong, China (People’s Republic); University of Reading, Reading, United Kingdom; Lingnan University, Hong Kong, Hong Kong, China (People’s Republic)

## Abstract

Multiple informal caregivers are often involved in dementia caregiving, yet little is known about how families collectively respond to care-related changes over time. Guided by family stress theories, this longitudinal study aimed to 1) examine changes in care demands and use of resources, and 2) identify predictors of adaptive family responses. Participants were 90 informal caregivers (22% spouses; mean age = 55.8 ± 12.4 years) of community-dwelling PLwD in Hong Kong. Data were collected via telephone interviews in 2021 and 2025, and analyzed using repeated measures and multiple regression. Over four years, PLwD showed significant declines in ADL, IADL, and health-related quality of life (QoL) (all p<.001). While the number of informal caregivers remained stable, there were increases in the use of foreign domestic helpers (38% to 52%), formal care services (39% to 62%), and informal care time (512 to 729 hours/month). Caregivers reported increased care burden (p<.001) and reduced health-related QoL (p=.005). After adjusting for functional decline, predictors of involving more informal caregivers were fewer members participating in care-related decision making (B=-0.716, p<.001) and higher care burden (B = 0.043, p=.007). Predictors of increased or sustained use of social services included fewer decision-making caregivers (OR = 0.341, p=.036), better relationship quality with PLwD (OR = 2.13, p=.048), and higher expectations for future caregiving needs (OR = 1.16, p=.028). Our findings suggest that families with enmeshed dynamics and misaligned care expectations may be at higher risk for maladaptive functioning, highlighting the need for early identification and targeted support programs in shaping adaptive responses to dementia care over time.

